# Preparation, characterization and immunological evaluation: canine parvovirus synthetic peptide loaded PLGA nanoparticles

**DOI:** 10.1186/s12929-015-0195-2

**Published:** 2015-10-20

**Authors:** Serap Derman, Zeynep Akdeste Mustafaeva, Emrah Sefik Abamor, Melahat Bagirova, Adil Allahverdiyev

**Affiliations:** Chemical and Metallurgy Faculty, Bioengineering Department, Yildiz Technical University, 34220 Istanbul, Turkey

**Keywords:** Canine Parvovirus, Vaccine, Antigen, Peptide, PLGA, Nanoparticle, Antigen delivery

## Abstract

**Background:**

Canine parvovirus 2 (CPV-2) remains a significant worldwide canine pathogen and the most common cause of viral enteritis in dogs. The 1 L15 and 7 L15 peptides overlap each other with QPDGGQPAV residues (7-15 of VP2 capsid protein of CPV) is shown to produce high immune response. PLGA nanoparticles were demonstrated to have special properties such as; controlled antigen release, protection from degradation, elimination of booster-dose and enhancing the cellular uptake by antigen presenting cells. Nevertheless, there is no study available in literature, about developing vaccine based on PLGA nanoparticles with adjuvant properties against CPV.

Thus, the aim of the present study was to synthesize and characterize high immunogenic W-1 L19 peptide (from the VP2 capsid protein of CPV) loaded PLGA nanoparticle and to evaluate their in vitro immunogenic activity.

**Results:**

PLGA nanoparticles were produced with 5.26 ± 0.05 % loading capacity and high encapsulation efficiency with 81.2 ± 3.1 %. Additionally, it was evaluated that free NPs and W-1 L19 peptide encapsulated PLGA nanoparticles have Z-ave of 183.9 ± 12.1 nm, 221.7 ± 15.8 nm and polydispersity index of 0.107 ± 0.08, 0.135 ± 0.12 respectively. It was determined that peptide loaded PLGA nanoparticles were successfully phagocytized by macrophage cells and increased NO production at 2-folds (*P < 0.05) in contrast to free peptide, and 3-folds (*P < 0.01) in contrast to control.

**Conclusion:**

In conclusion, for the first time, W-1 L19 peptide loaded PLGA nanoparticles were successfully synthesized and immunogenic properties evaluated. Obtained results showed that PLGA nanoparticles enhanced the capacity of W-1 L19 peptide to induce nitric oxide production in vitro due to its adjuvant properties. Depend on the obtained results, these nanoparticles can be accepted as potential vaccine candidate against Canine Parvovirus. Studies targeting PLGA nanoparticles based delivery system must be maintained in near future in order to develop new and more effective nano-vaccine formulations.

## Background

Canine parvovirus (CPV) is a small, non-enveloped, autonomously replicating Single-strained DNA virus [1], remains a significant worldwide canine pathogen [2], is the cause enteric and myocardial disease in dogs [3, 4]. In experimentally affected dogs, mortality without treatment has been reported as high as 91 % [2, 5]. The two peptides overlap each other with the sequence QPDGGQPAV residues (7 to 15 of VP2) 1 L15 (MSDGAVQPDGGQPAV) and 7 L15 (QPDGGQPAVRNERAT), have different potencies in inducing virus-neutralizing antibodies, produce good immune response in mice and immunogenic in several animal species [6, 7]. Therefore, different approaches, particularly using these peptide sequences, are available for developing synthetic peptide based vaccines against Canine Parvovirus [6–9]. However, synthetic peptides when used as a vaccine, without a delivery system have been shown to be ineffective due to its rapid degradation by proteases, along with its poor cellular uptake and immunogenicity [10]. In order to elicit a higher immune response and improve the efficiency of peptide-based vaccine, it is generally necessary to use a carrier system such as protein, polymer or nano-micro particles.

Nanoparticle based antigen delivery system is a rapidly developing area within nanotechnology. Especially nano sized particular system based on biodegradable polymers offer potential solution to disadvantages of the current vaccines [11]. Poly(DL,lactic-*co*-glycolic acid) (PLGA) is approved by Food and Drug Administration (FDA) and widely used copolymer for nanoparticular delivery system, owing to its biodegradability and biocompatibility [12]. Encapsulation of vaccine antigens using PLGA nanoparticles provides several advantage over the other antigen delivery systems, such as; (i) antigen can be controlled released over a longer period [13], (ii) antigen can be protected against degradation in the presence of proteolytic enzymes [14], (iii) eliminated the need for booster dosed [15], (iv) enhance the antigen cellular uptake by antigen presenting cells (APC) [13, 14]. Moreover, their submicron size and their large specific surface area favor their adsorption compared to larger carriers [16]. According to these significant properties of PLGA polymer were studied in order to develop new peptide based vaccine delivery systems against infectious disease such as Bacillus anthracis [11], Hepatitis B [17, 18], Chlamiydia Trachomatis [19], and also against allergic asthma [20], melanoma cancer [21]. However, to our knowledge, there is no study available in literature, in regards to develop vaccines based on PLGA nanoparticles with adjuvant properties against CPV.

The main goal of the present study was to synthesize and characterize PLGA nanoparticle loaded with high immunogenic W-1 L19 peptide sequences from the VP2 capsid protein of CPV and to evaluate their in vitro immunogenic activity. For this purpose, loading capacity, encapsulation efficiency, antigen release and morphological investigation of the nanoparticles were conducted. Additionally, in vitro cytotoxicity of nanoparticles was investigated on J-774 cell lines and finally potency of PLGA nanoparticles to induce NO production at non-toxic concentrations were evaluated in macrophages.

## Methods

### Materials

The water-soluble synthetic peptide representing W-1 L19 from the VP2 capsid protein of Canine Parvovirus (W-MSDGAVQPDGGQPAVRNERA) and the non-immunogenic scrambled peptide (WMSDGAVQPDGGQPAVRNERA) were synthesized via solid phase peptide synthesis method by Caslo Laboratory ApS (Denmark). Tryptophan (W) amino acid was also added to the N-terminus of peptide sequences in order to provide UV-spectral analysis. PLGA (lactide:glicolide = 50:50; inherent viscosity 0.45-0.60 dL/g, Mw ~ 38-54 kDa P50/50), 3-(4,5-dimetil triazol-2-il)-2,5-difeniltetrazoliumbromid (MTT), Fluorescein isothiocyanate (FITC), dimethyl sulfoxide (DMSO), and polyvinyl alcohol were purchased from Sigma Aldrich (St. Louis, USA), dichloromethane (DCM) was purchased Ridel de Haen. Mouse J774 macrophage cell line was obtained from Histology and Embryology Department, Istanbul University, Istanbul, Turkey. Ultra-pure water was obtained from Millipore MilliQ Gradient system.

### Methods

#### Preparation of polymeric nanoparticles

Canine Parvovirus W-1 L19 peptide was encapsulated as an antigen in PLGA nanoparticles by a modified water/oil/water double emulsion solvent evaporation method [22]. Briefly, primary emulsion between internal aqueous phase containing peptide (5 mg/ml) and organic phase (75 mg/ml PLGA in dichloromethane) was prepared by sonication (55 W, amplitude of % 50, 2 min) (Bandelin Sonopuls, Germany) over an ice bath. Thereafter, the resulting primary emulsion (w/o) was added drop wise to external aqueous phase containing 4 ml PVA (% 2.5 w/v) and emulsified in an ice-water bath to form the double emulsion (w/o/w). The emulsifications were carried out using micro tip probe sonicator set at 55 W of energy output (Bandelin-sonopuls) for 2 min in an ice bath. The double emulsion (w/o/w) was diluted in 80 ml PVA (% 0.5 w/v) solution and the emulsion was stirred overnight on a magnetic stirrer plate at room temperature for evaporation of organic phase. The resulting particles were collected by centrifugation at 10.000 x g for 20 min (Sartorius-Biofuge), washed three times with ultra-pure water to remove excess PVA and then lyophilized. We also prepared FITC loaded nanoparticles, for morphological investigation with fluorescence microscopy and in vitro cellular uptake study. Fluorescent nanoparticles were fabricated in a similar method where FITC was used in place of peptide. An equivalent volume of ultra-pure water was similarly encapsulated in P50/50 to serve as a control (Free NP). All lyophilized nanoparticles were stored at −80 °C until used.

#### Encapsulation efficiency and peptide loading capacity

Peptide encapsulation efficiency (EE) was detected via indirect quantification methods by using UV-Vis Spectroscopy at 280 nm. EE was determined by measuring the concentration of free peptide in supernatant which obtained from the ultracentrifugation of nanoparticles. The peptide concentration in the supernatant was determined by comparing the concentration to a previously constructed standard calibration curve. The concentration of loading peptide was calculated indirectly by calculating the differences between the initial concentrations of the peptide used (5 mg/ml) and the concentration of free peptide in supernatant.

The peptide encapsulation efficiency (EE) and the peptide loading capacity (LC) were calculated using the formulas given below:

1 $$ \mathrm{E}\mathrm{E}=\left(\mathrm{A}\hbox{-} \mathrm{B}/\mathrm{A}\right)\times 100 $$

2 $$ \mathrm{L}\mathrm{C}=\left(\mathrm{A}\hbox{-} \mathrm{B}/\mathrm{C}\right)\times 100 $$

Where A is the total peptide amount, B is the free peptide amount, and C is the quantified nanoparticle weight [19]. A standard calibration curve of the absorbance as a function of peptide concentration was studied at 280 nm. All measurements were performed in triplicate.

#### Atomic force microscopy (AFM), scanning electron microscopy (SEM), fluorescent microscopy (FM)

Both atomic force microscopy (AFM) and scanning electron microscopy (SEM) were used to ascertain surface morphology and size of nanoparticles (free and peptide loaded). AFM (Shimadzu SPM 9600, Japan) studies were performed as previously described [23]. About 5 μl each of nanoparticle solution were dropped to freshly cleaved 1 cm^2^ mica surface and incubated for 5 min. Mica surface was rinsed with ultra-pure water and dried for 20 min, the morphological analysis was performed by dynamic mode in 2-dimensional (2-D) and 3- dimensional (3-D).

SEM was used to verify uniformity of nanoparticle shape and size as previously described [24]. The fabricated nanoparticles were dropped onto black carbon tape with a double-side. After that, they were vacuum-coated with a platinum mixture for 45 s and morphologically analyzed with a FE-SEM (CamScan Apollo 300 Field-Emission SEM, UK) at 20 kV.

#### Fourier transform infrared (FT-IR) spectrometry

Infrared Spectroscopy of the samples was performed in IR-Prestige 21 FTIR Spectrophotometer (Shimadzu, Japan). FT-IR spectra were recorded for P50/50, W1L-19 peptide and peptide loaded nanoparticle in universal attenuation total reflectance (ATR) mode. The measurement range was 4000–750 cm^−1^, scan number for per sample was 16, and resolution was 4 cm^−1^.

#### Particle size, zeta potential and polydispersity index (PdI)

The intensity size distribution, the Z-average (Z-Ave), and PdI of nanoparticles were performed by using dynamic light scattering technique using a Zetasizer (Zetasizer Nano ZS, Malvern, UK) instrument equipped with 4.0 mV He-Ne laser (633 nm). Measurements were carried out at 25 ± 0.1 °C with using 0.8872 cP of viscosity and 1.330 of refractive index for the solutions. The number of runs and run durations were chose as automatically. Electrophoretic light scattering (ELS) is used for zeta potential (ζ) measurement of particles and carried out in the folded capillary cell at 25 ± 0.1 °C. The measurements were performed with the following parameters: viscosity, 0.8872 cP; dielectric constant, 79; f(ka), 1.50 (Smoluchowski). The measurement durations and voltage selections were set to automatic mode.

All samples were prepared by diluting with phosphate buffer saline (PBS), filtered with a 0.20 μm RC-membrane filter (Sartorius) before measurement, and all measurements were performed three times.

#### In vitro peptide release

The release of the W-1 L19 peptide in vitro from the peptide loaded nanoparticle were determined following the method of Dixit et al. [25]. Briefly the peptide loaded nanoparticle aliquots suspended in PBS (pH 7.4) with % 0.01 sodium azide and the suspension were incubated at 37 °C in a shaking incubator (60 rpm). At predetermined time intervals (6 h, 12 h, 1, 3, 7, 14, 21, 28, 35, 42 days), tubes were centrifuged, and the supernatants were collected followed by resuspension pellet in fresh PBS. The peptide concentration in the supernatant was determined with UV-Vis Spectroscopy at 280 nm by comparing the concentration to a previously constructed standard calibration curve.

#### Morphological changes depend on the time

In order to examine morphological changes depending on the time (during the degradation of nanoparticles), the nanoparticle suspension in PBS were incubated at 37° and samples were analyzed in 30 and 60 days with AFM.

#### Cell viability assay

Cell viability assays of nanoparticles and peptides were performed on J774 cell lines by MTT method. Briefly, 3 × 10^4^ cells/ml were seeded into microplates and were incubated overnight. After incubation, different concentrations of nanoparticles and peptides ranging from 1 mg/ml to 0.01 mg/ml in PBS were added onto cells. Following to 48 h incubation at 37 °C, 10 μl MTT reactant 3-(4,5-Dimethyl-2-thiazolyl)-2,5-diphenyl-2H-tetrazolium bromide (10 mg/ml) were included into all wells of microplates in order to assess susceptibility of cells against agents. When formazan crystals that are signature of viability were detected, these crystals were dissolved by DMSO. Then colorimetric density in microplates was read by using a Microplate Reader at 570 nm.

### Cellular uptake study

In order to observe uptake of nanoparticles into macrophages, 5 × 104 cells/ml were seeded into each wells of a 6 well-plate. Following to one night incubation, nanoparticles at the concentrations of 0.5 mg/ml, which was detected as non-toxic concentration for macrophages were put in each wells of the plate. Cellular uptake was visualized by a florescence microscope thanks to fluorescein molecule which was embedded into nanoparticles. Photographs related to uptake were taken by Olympus C-5050 digital camera.

### Quantification of nitrix oxide (NO) level

In experiments, in order to evaluate nitric oxide production following to nanoparticle and peptide formulations, we used non-toxic concentration (500 μg/ml) of both nanoparticles and peptide. In concentrations higher than 500 μg/ml, there was a sharp decline in viability values of J774 cells in regardless of nanoparticle and peptide. Therefore, a concentration of 500 μg/ml was chosen both nanoparticles and peptides. For this experiment, 5 × 10^4^ J774 macrophage cells were seeded into 96 well microplate. After overnight incubation, macrophage cells were exposed to free NP, peptide loaded NP and peptide with the concentrations of 500 μg/ml, while only PBS was used in control group. Following to 48 h incubation, supernatants above the cells were picked up and 50 μl of supernatants from each group were transferred to another microplate. In order to observe colorimetric reaction and evaluate nitric oxide production, 50 μl of griess reactive was transferred to all wells of new microplate. On the other side, different concentrations of nitride were used as control. After 30 min incubation at room temperature, microplate was read at 540 nm absorbance by using a Microplate-Reader.

### Statistical analysis

All experiments were repeated at least three times in triplicate wells. Data were expressed as mean ± standard deviation. All statistical analyses were performed using SPSS 15.0 [26]. Non parametric analysis with Mann-Whitney *U*-test was carried out on the data of the biological variables to examine differences between the groups. P value less than 0.05 (*p* < 0.05) was accepted as significant.

## Results and discussion

### Encapsulation efficiency and loading capacity

The w/o/w double emulsion solvent evaporation method was used for fabrication of W-1 L19 peptide loaded PLGA nanoparticles. The ratio of aqueous phase (containing W-1 L19 peptide) to organic phase (containing PLGA) was kept low for production of nano-size particles as this ratio directed affects the particle size. First, we determined process yield, peptide loading capacity, and encapsulation efficiency. Lyophilized nanoparticles were weighed and yield of the process was estimated to be 81.2 ± 3.1 %. Peptide loading capacity and encapsulation efficiency were determined by measuring the concentration of free peptide in supernatant which obtained from the ultracentrifugation of nanoparticles. Our results from indirect methods employed for calculate the EE and LC and they were found 85.3 ± 2.2 % and 5.26 ± 0.05 % respectively.

### Physicochemical properties of nanoparticles

**Fig. 1:**
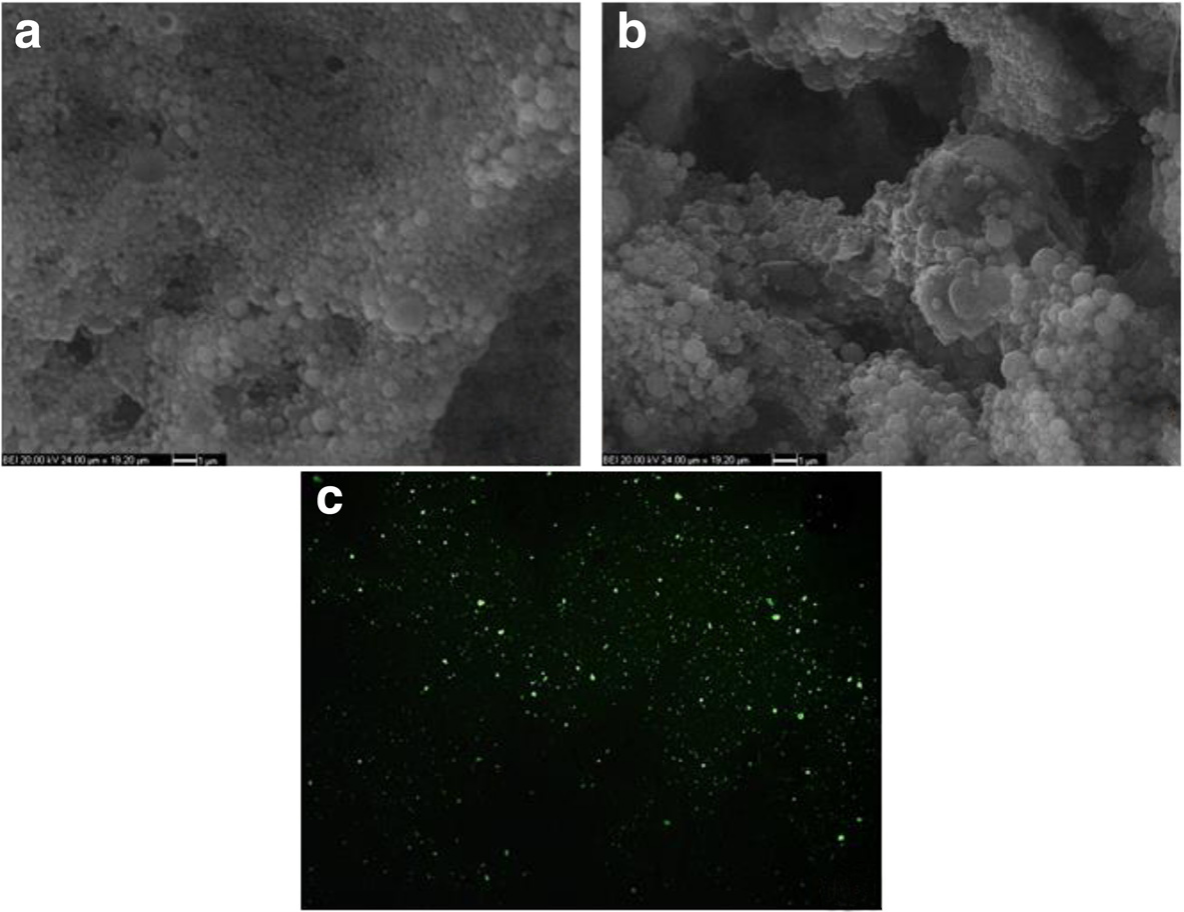
Scanning electron microscope image of free (**a**) and peptide loaded (**b**) PLGA nanoparticles (Magnification 5000x). Fluorescence microscope (40×) image of FITC + peptide loaded nanoparticles (**c**)

**Fig. 2:**
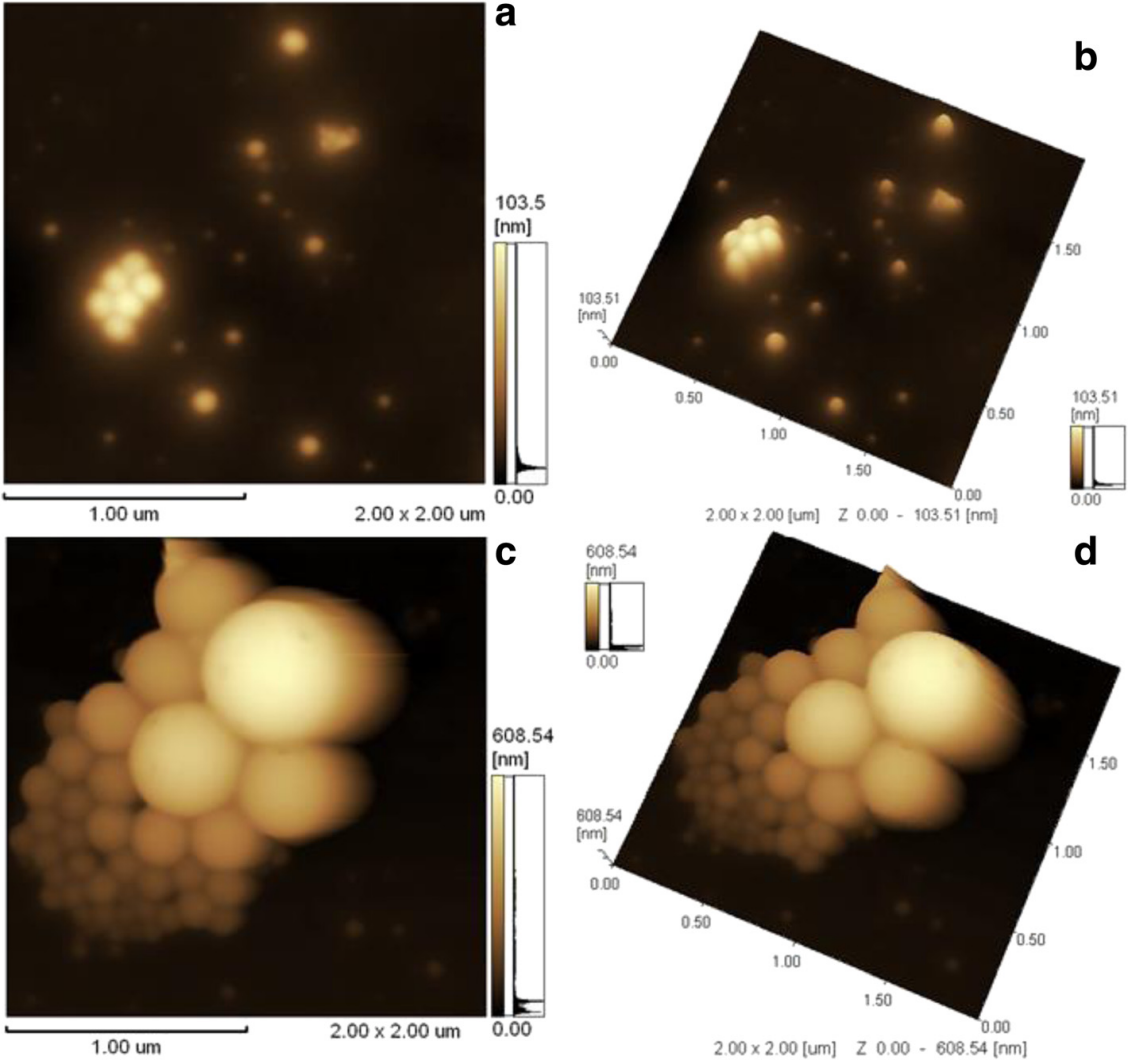
2-Dimensional (**a** and **c**) and 3-Dimensional (**b** and **d**) atomic force image of free (**a** and **b**) and peptide loaded (**c** and **d**) PLGA nanoparticles

The nanoparticles have uniform size distribution, smooth surface and spherical shape is considered the best for an antigen depot (adjuvant) and to provide a controlled release formulation [11, 27, 28]. SEM (Fig. 1) and AFM images (Fig. 2) showed that the fabricated nanoparticles were typically spherical in shape and smooth surfaced with no cavities overall in most of observed particles.

### Fourier transform infrared (FT-IR) analysis

**Fig. 3:**
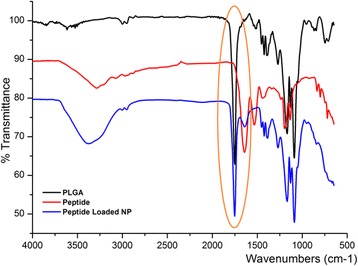
Furier transforms-infrared spectroscopy studies of PLGA (50/50) polymer, free nanoparticle and peptide loaded nanoparticles

The FT-IR spectra of peptide, PLGA and peptide loaded NPs were presented in Fig. 3. PLGA sample showed peaks such as aliphatic –CH stretching (2850–2950 cm^−1^), carbonyl –C = O stretching (1700–1800 cm^−1^ strong and narrow), C–O stretching (1050–1250 cm^−1^). The free peptide sample showed the main peaks contributed by the functional groups of molecules such as carboxylic acid O-H stretching (2500–3000 cm^−1^, board), overlapped amine and amide N-H stretching (3300–3500 cm^−1^ and 3500–3700 cm^−1^) and amide C = O stretching (1600–1690 cm^−1^). However, in the FTIR spectra for peptide loaded nanoparticles, the major peak of peptide at 1600–1690 cm^−1^ was significantly lowered. This indicated that the peptide was encapsulated in PLGA nanoparticles successfully.

### Particle size, zeta potential and PdI measurements

**Fig. 4:**
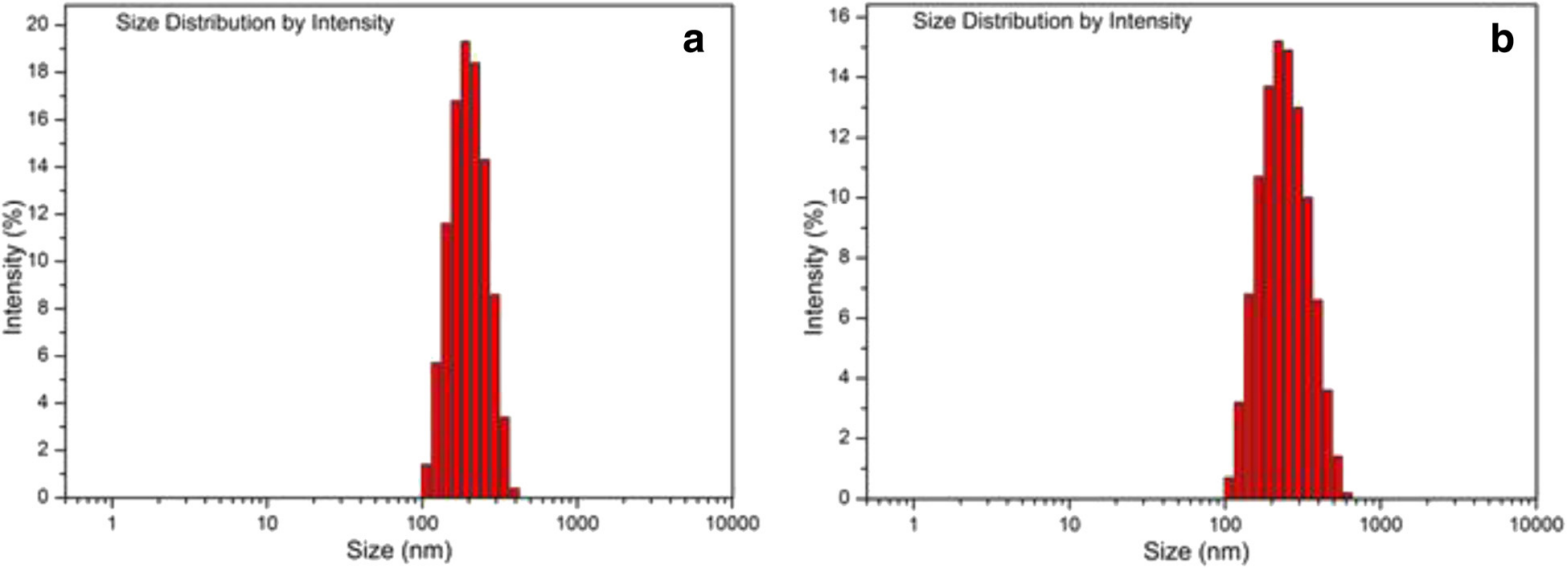
Size distribution analysis of free (**a**) and peptide loaded (**b**) nanoparticles

The size and size distribution of nanoparticles were investigated by dynamic light scattering technique. Particle size and surface characteristics, plays an important role in vitro and in vivo [20], such as determining the level of cellular and tissue uptake and immun response [27–30]. Also, zeta potential is one of the most important particle properties affecting particle stability. Figure 4 shown intensity size distribution of free (A) and peptide loaded (B) nanoparticles, respectively. Table 1 represent Z-Ave, PdI and Zeta Potential of free and peptide loaded particles. As it could be observed in Fig. 4 and Table 1 that, although peptide encapsulation had significant effect on the particle size, on zeta potential hadn’t significant effect. In the present study, Z-Ave of peptide loaded nanoparticles was approximetely 221.7 ± 15.8 nm (d. nm), which is in the good size range for cellular uptake [31]. Similarly, the Z-Ave of free nanoparticles fabricated in this study was 183.9 ± 12.1 nm (d. nm).

**Table 1:** Size distribution, zeta potential and PdI values of nanoparticles

Nanoparticles	Z-Ave (d.nm)	PdI	Zeta potential (mV)
Free nanoparticle	183.9 ± 12.1	0.107 ± 0.08	−36.8 ± 3.5
Peptide loaded nanoparticle	221.7 ± 15.8	0.135 ± 0.12	−35.1 ± 2.9

The free and peptide loaded nanoparticles showed a negative surface charges of around 36.8 ± 3.5 and 35.1 ± 2.9 mV, respectively which means that they were stable in dispersion state (Table 1). Further, PdI values are 0.107 ± 0.08 and 0.135 ± 0.12 for free and peptide loaded nanoparticle, respectively. The nanoparticles exhibited a relatively narrow PdI (less than 0.15) indicating the monodisperse formulation, which is useful for treatment effect [20].

### In vitro release study of peptide loaded nanoparticles

**Fig. 5:**
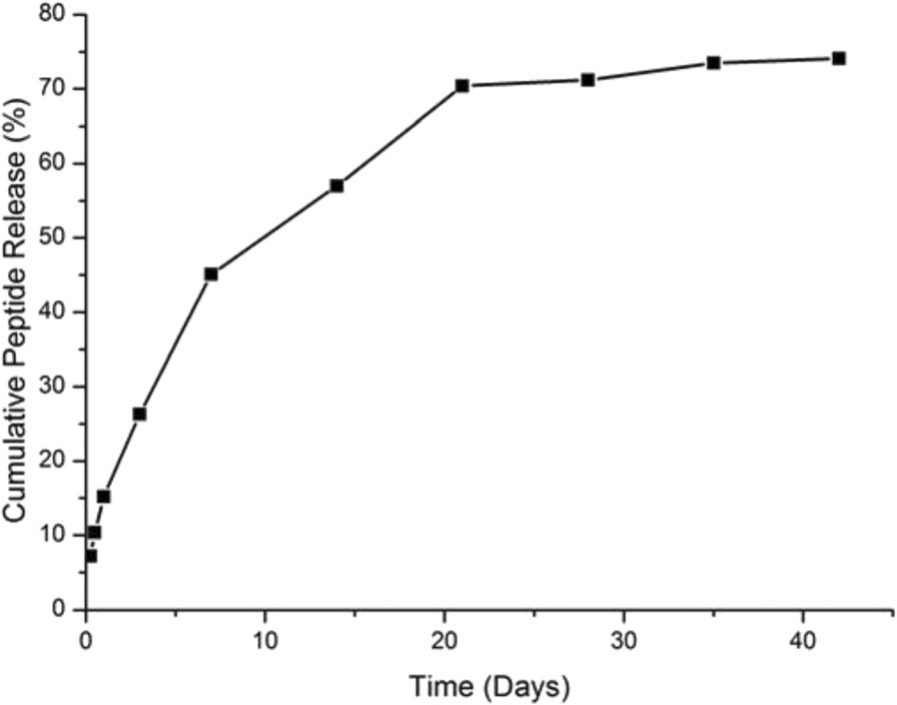
In vitro cumulative release of W1L-19 peptide from encapsulated PLGA nanoparticles in PBS incubated at 37 °C

The in vitro controlled release study was evaluated in PBS at pH 7.4. As shown in Fig. 5, a triphasic release pattern could be seen at this condition. In the first 7 days, a burst release showed with approximately 45 % of peptide was released. For the second part of kinetic, peptide release reached a plateau in the next 14 days, with a cumulative release of 70.4 %. Additionally, almost 75 % of peptide was cumulatively released form nanoparticles within 42 days and no further release was observed (Fig. 5). Encapsulation to PLGA provided a slow release of peptide [32], which is an interesting attribute property for a vaccine candidate as this may reduce the immunization number as well as enhancing the presentation of peptide to Antigen presenting cell (APC) [19].

### Morphological changes depend on the time

**Fig. 6:**
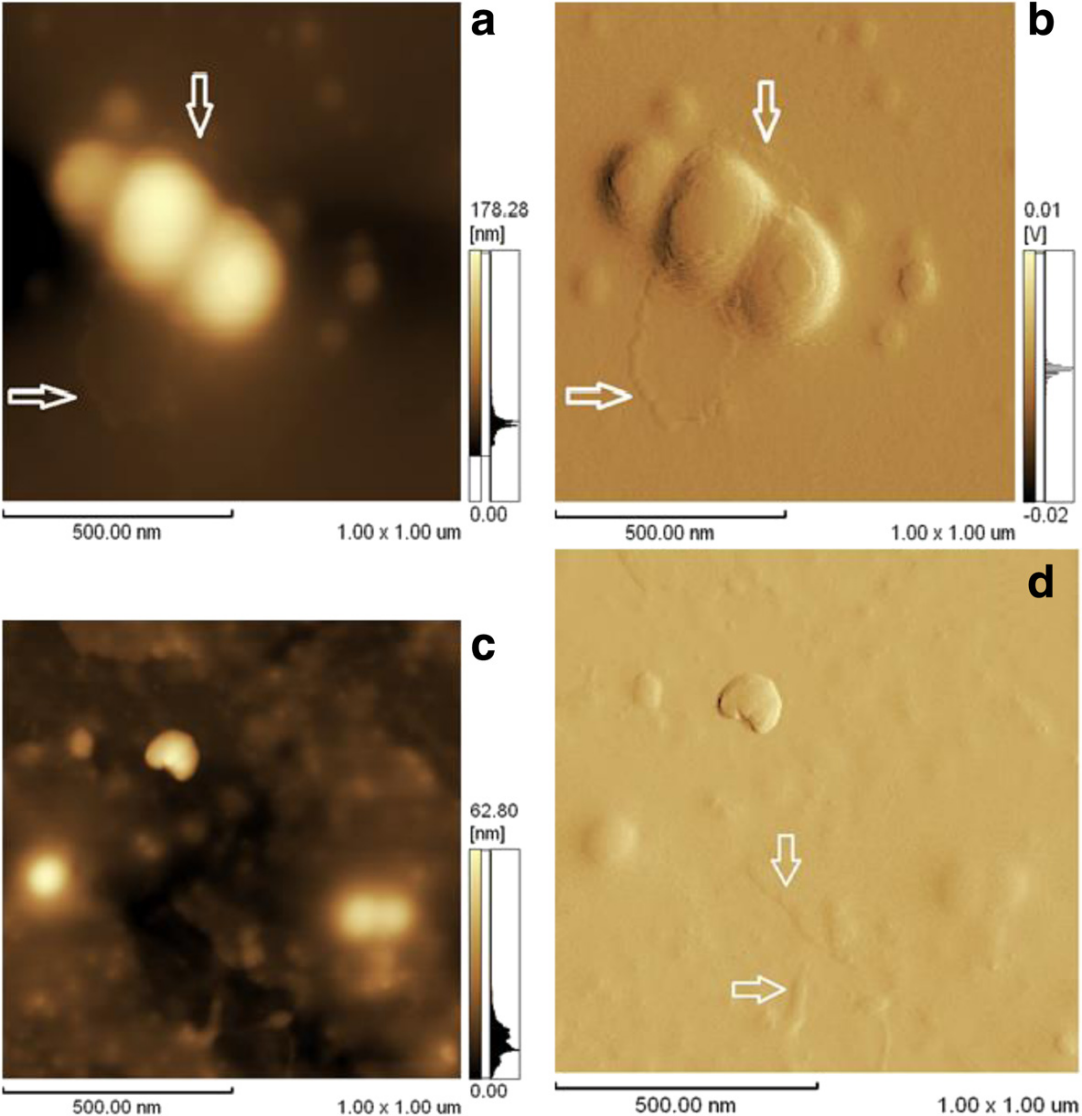
2-Dimensional (**a** and **c**) and deflection (**b** and **d**) AFM image of nanoparticles after 30 days (**a** and **b**) and 60 days (**c** and **d**) incubation in PBS at pH 7.4

Also we examined morphological changes of nanoparticles in PBS at pH 7 during the degradation in 30th and 60th days with AFM. Time dependent-morphological changes during degradation period in 30 days and 60 days were shown in Fig. 6a, b, c and d, respectively. PLGA nanoparticles show morphological changes after the 30 day period and significant changes for 60th days.

Especially AFM views related to morphology of nanoparticles at the day 60 demonstrated that nanoparticles lost their spherical shapes and this can be explained by the hydrolysis of PLGA nanoparticles. In Fig. 6c and d, the area outside of nanoparticles within release medium were observed as “foggy”. This turbidity may be explained by hydrolysis of nanoparticles and release of peptide antigen to the medium.

### Cell viability assay

**Fig. 7:**
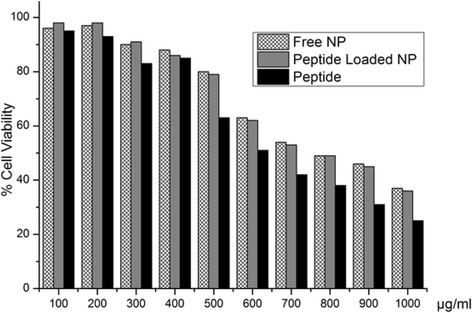
Cytotoxicity of nanoparticles and peptide on J774 macrophage cells

According to the results (Fig. 7), concentrations between 100 μg/ml and 500 μg/ml are found as non-toxic on J774 macrophage cells for free and peptide loaded NP formulations and peptides. In concentrations higher than 500 μg/ml there were sharp declines in viability values of macrophages. When the cells were morphologically analyzed, it was also observed that macrophages that were exposed to concentrations higher than 500 μg/ml lost their stabilities and amounts of formazan crystals that are signs of cellular viability decreased, substantially in contrast to control group. On the other side, macrophages that were exposed to formulations at 500 μg/ml, stayed compact and amounts of formazan crystals within the cells were close to control. IC50 values of free NP, peptide loaded NP and peptides were assessed as 750, 740 and 650 μg/ml, respectively. Due to these results, concentration of 500 μg/ml was selected for further nitric oxide evaluation and cellular uptake assays, since this concentration was considered as the highest nontoxic concentration.

### Cellular uptake

**Fig. 8:**
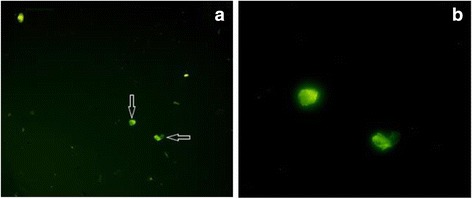
Fluorescence microscope images with 10 × (**a**) and 40 × (**b**) magnification of J774 macrophages exposed to FITC + peptide loaded nanoparticles for 24 h at 37 °C under 5 % CO2 in complete media

When cellular uptake of nanoparticles was observed by using fluorescence microscope, it was detected that nanoparticles were easily phagocyted into macrophages and located in their vacuoles independent from the concentrations of nanoparticles. Uptake of fluorescence loaded nanoparticles into macrophages was demonstrated in Fig. 8. From the figure it was also seen that macrophages protect their compact structure and no cytotoxic effect was available. Interaction between nanoparticles and macrophages shows that nanoparticles can easily penetrate into phagocytes and can present antigens that they are carrying for activating macrophages to compose immunogenic responses.

### Quantification of nitrix oxide (NO) Level

**Fig. 9:**
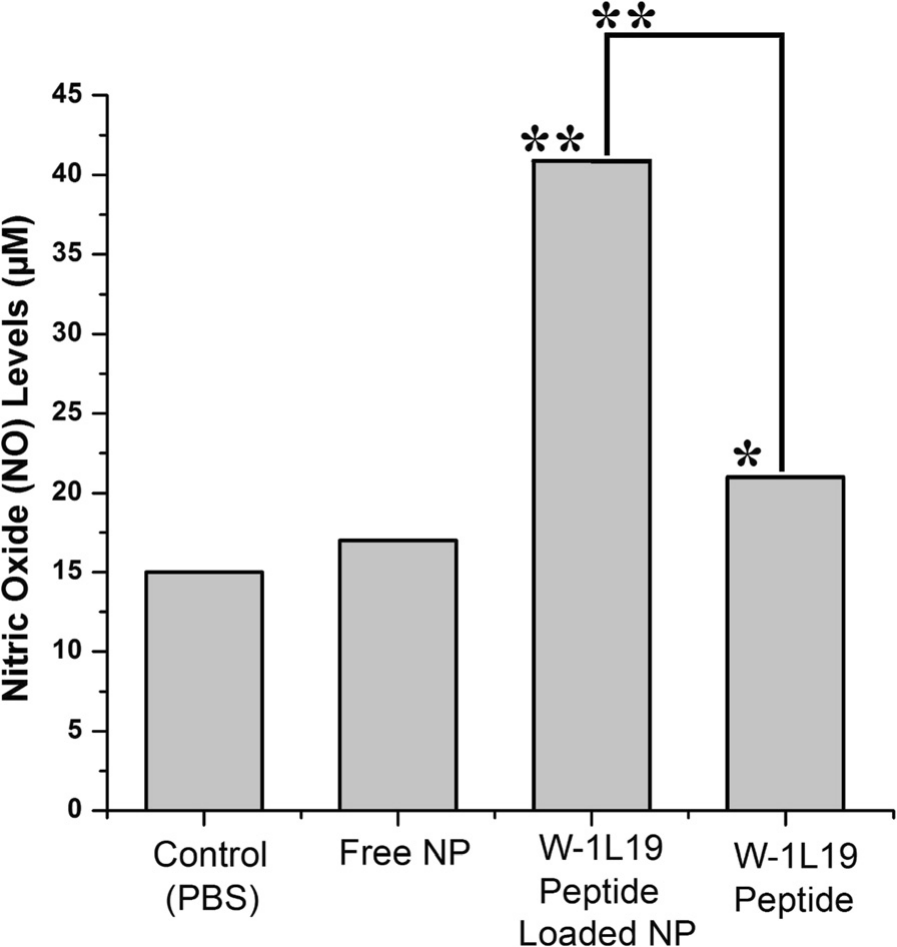
Nitric oxide production of J774 macrophage stimulated with free NP, peptide loaded NP and peptide. Significant differences in production of nitric oxide from stimulation are marked with asterisks. Significance levels of *P < 0.05, **P < 0.01, by non-parametric Mann-Whitney *U*-test

According to the results, it was observed that a peptide loaded NP formulation induced nitric oxide production of macrophage cell much higher than peptide and free NP use alone. In group that was exposed to peptide loaded NPs, macrophages produced approximately 3 fold nitric oxide in contrast to macrophages in control and experiment group that was exposed to free nanoparticles that did not contain any antigenic molecules. Similarly, macrophages exposed to peptide loaded NPs produced approximately 2 fold nitric oxide in contrast to macrophages exposed to only peptide antigen (Fig. 9). Since nitric oxide that are produced by macrophages is one of the important factors of immune system in regards to fighting against microorganisms, determination of enhanced nitric oxide levels in macrophages that are induced by peptide antigen containing nanoparticles can be a corner step in activating immune system and developing new vaccine candidates based on delivery systems.

## Conclusion

Poly(DL,lactic-*co*-glycolic acid) is approved by Food and Drug Administration (FDA) and widely used copolymer for nanoparticular delivery system, owing to its biodegradability and biocompatibility [12]. In the previous studies demonstrate that, PLGA nanoparticles have adjuvant effect for various vaccine antigens [17, 33–35]. However, we could not find any study in literature investigating immunogenic features of PLGA nanoparticles as adjuvants against Canine Parvovirus.

In this study, for the first time, W-1 L19 peptide loaded PLGA nanoparticles were successfully synthesized by using water/oil/water double emulsion solvent evaporation method. Results of particle characterization with SEM, AFM, FT-IR and zetasizer demonstrated that synthesized particles were nano-sized, narrow sized distributed and smooth spherical shaped. Moreover, controlled release of W-1 L19 peptide from the particles were observed under physiological pH (7.4). According to biocompatibility tests of nanoparticles that were maintained on J774 cell lines, non-toxic concentrations of W-1 L19 peptide loaded PLGA nanoparticles were found and their high immunogenic features were determined by evaluation of nitric oxide amounts in macrophages cells.

As it is known, in vaccine delivery researches based on PLGA nanoparticles, especially 200-500 nm ranged particles are preferred [11, 27, 28, 36, 37]. That’s why at these dimensions, PLGA nanoparticles can easily activate dendritic cells, antigen specific T helper cells and cytotoxic T lymphocyte cells in order to generate high humoral and cellular immune response, they can be endocytosed by antigen presenting cells (APCs) as well [11, 14, 37].

In the present study, we encapsulated Canine Parvovirus W-1 L19 antigenic peptide to PLGA (50:50) nanoparticles by double emulsion solvent evaporation method [22] with small modifications. PLGA nanoparticles were produced with 5.26 ± 0.05 % loading capacity and high encapsulation efficiency with 81.2 ± 3.1. Additionally, it was evaluated that free NPs and W-1 L19 peptide encapsulated PLGA nanoparticles have Z-ave of 183.9 ± 12.1 nm and 221.7 ± 15.8 nm, respectively. It can be thought that synthesized nanoparticles are small enough to interact with APCs and induce cellular and humoral immune response.

Zeta potential is the essential particle characteristic and affecting particle stability, all of studies about zeta potential of PLGA nanoparticle resulted that PLGA nanoparticles which were prepared with PVA as a surfactant has a negative surface charge [14, 19, 20, 31, 38, 39]. Similarly in our study, the zeta potential of free NPs and peptide loaded NPs was −36.8 ± 3.5 mV and −35.1 ± 2.9 mV respectively, indicating a high stability due to the high repulsion between nanoparticles.

Characterization of W-1 L19 peptide encapsulated PLGA nanoparticles with AFM and SEM exhibited that synthesized nanoparticles were smooth surfaced and spherical in shape. In several studies, uses of smooth and spherical nanoparticles were suggested as well adjuvant activity for antigens and there was no requirement to apply booster doses of vaccines since they provide opportunity to controlled release by degradation of PLGA nanoparticles [11, 27, 36]. In general biodegradation of nanoparticulate vaccine delivery systems are investigated by evaluating release kinetics. For the first time, in this study, we visualized nanoparticulate system for 30 and 60 days in physiological conditions (pH 7.4 and 37 C) by using AFM. According to AFM images, we determined that nanoparticles protected their spherical shapes at the end of 30^th^ days, while nanoparticles were totally lost their compact structures at the end of 60^th^ days showing that PLGA nanoparticles released high amounts of W-1 L19 antigens to the medium.

In studies targeting development of vaccine delivery systems, long-time release of antigens from nanoparticles is crucial as they can provide long-term-protection against diseases [11, 14, 40]. This may also reduce quantities of immunization process and increase the antigen presentations to APCs [40]. Depending on their biodegradable features which lead to long-term controlled release as well as biocompatibility, PLGA nanoparticles have been widely studied in vaccine development especially against infectious diseases [11, 18, 25] and cancer [21, 41–43]. Taha et al exhibited that PLGA nanoparticles caused the 20 % release of Major Outer Membrane Protein (MOMP) at one day and 48.6, 70 and 100 % of antigen were released at first, second and third week, respectively [19]. Manish et al studied on protective efficacies of Immunogenic Domain 4 of Protective Antigen (PAD4) loaded PLGA nanoparticles against *Bacillus anthracis*. This group demonstrated that 50 % of PAD4 antigens released from nanoparticles during first 24 h. Totally 75 % of PAD4 antigens released at the end of 4 weeks [11]. In the another study, Primard et al, prepared multifunctional PLGA nanoparticles by encapsulating an immunomodulator Imiquimod (IMQ) and BSA as an antigen in order to target Toll-like Receptor 7. In these study, it was shown that PLGA nanoparticles rapidly released % 40 of IMQ at 24 h [39]. As it is clearly seen, in most of studies, PLGA nanoparticles showed high burst-release kinetics which is identified as high amounts of antigens’ release in 24 h [11, 19, 39]. However, in some studies it was pointed out that high burst release especially in 24 h is not preferred since it leads to low T cell response and antigen encapsulated nanoparticles with low burst release features may demonstrate better vaccine activity. For that reason, Silva and colleagues synthesized 24-residue long synthetic antigenic peptide of Ovalbumin (OVA24) peptide encapsulated PLGA nanoparticles with w/o/w double emulsion method and studied on diminishing burst release of antigens from nanoparticles by changing first and second emulsion medium. These group compared low (<10 %) and high (75 %) burst release in terms of immunogenic properties and found out that low burst release resulted in higher T cell response [14]. Similarly, in our study, only 7 % of antigens were released at first 24 h which indicates sustained slow release. Our results overlapped with similar studies [14, 44–46] since antigen release from PLGA nanoparticles were shown to be biphasic release character. These results show that our synthetized particles might be attractive candidate for further vaccine studies.

One of the most important properties of PLGA nanoparticles is improving biocompatibility and bioavailability of biologically active molecules such as peptides, drugs, proteins etc [47]. In several studies, it has been shown that encapsulation of antigenic molecules into PLGA nanoparticles decreased their toxicity as well as enhancing their bioavailability [11, 14, 17–21, 25, 38, 39, 48]. Our findings demonstrated that IC50 values of free NP, peptide loaded NP and peptides were assessed as 750, 740 and 650 μg/ml, respectively. In all of studied concentrations, encapsulation of peptides into PLGA nanoparticles increased applied dosages of peptides since viability amounts of macrophages that were exposed to free peptides were lower than macrophages that were exposed to peptide loaded PLGA nanoparticles [11, 14, 17–19, 21, 39, 48]. This is related to special features of PLGA nanoparticles that enhance biocompatibility of used antigens. For nitric oxide production and cellular uptake studies, concentrations of 500 μg/ml were chosen as macrophage cells stayed compact and showed no cytotoxicity on J774 macrophage cells, while at concentrations higher than 500 μg/ml macrophage cells started to lose their compact morphology and their viability values decreased sharply.

As it is known, phagocytosis of a particle into macrophage cells is influenced by the size, shape and surface properties. In various studies, it was demonstrated that PLGA micro/nanoparticles were internalized by macrophages with different pathways such as phagocytosis (particle size: 0.5 μm-10 μm) macro-pinocytosis (particle size: 100 nm-5 μm), clathrin-mediated pinocytosis (particle size: approximately 120 nm), caveolin-mediated pinocytosis (particle size: approximately 80 nm), clathrin- and caveolin-independent pinocytosis (particle size: approximately 50 nm) [49–51]. However, accurate uptake mechanisms of PLGA nanoparticles have not been understood clearly, anymore. According to literature data, we can think that our nanoparticles which was sized as 221.7 ± 15.8 nm, may internalize into macrophages by using macro-pinocytosis pathway [52].

Nitric Oxide (NO) is one of the most important immune-effector molecules in the body, playing role in host defense in bacteria, fungi, parasites and viruses [53, 54]. NO can enhance immune response again infections by stimulating cytokine production and leading macrophages to kill intracellular pathogens [55]. Therefore, in vaccine studies, determination of increased NO levels following to exposure to applied immunogenic molecule is substantial since it is the sign of augmented immune response. Accordingly, in our study, we studied on production of NO by macrophages after treatment with control (PBS), free PLGA nanoparticles, peptide alone and peptide loaded PLGA nanoparticles. Due to the results, it was determined that peptide loaded PLGA nanoparticles increased NO production at 2-folds (∗*P* < 0.05) in contrast to free peptide, and 3-folds (∗*P* < 0.01) in contrast to control and free PLGA nanoparticles. The significant difference especially between peptide loaded nanoparticles and free nanoparticles can be explained by high adjuvant features of PLGA nanoparticles [27]. We think that PLGA nanoparticles enhanced antigenicity of peptides due to their special properties while it did not stimulate any immune response as a good adjuvant need to do. This implicated that peptide loaded PLGA nanoparticles were good at enhancing immune response and may also stimulate the other immunological pathways.

In conclusion, to our knowledge this is the first study to synthesis and characterization and in vitro evaluation of W-1 L19 peptide encapsulated PLGA50:50 nanoparticles and its immunostimulating effect on J774 Murine macrophage-like cells. Both particles size distribution, zeta potential and sustained slow release of antigenic peptide from nanoparticles together with accomplishments to induce significantly higher NO production than free peptide, propose that nanoparticular system can be interesting vaccine candidate against Canine parvovirus infections. However, we think that much more efforts must be performed especially on the subject of in vitro stimulation of immune response following to W-1 L19 peptide encapsulated PLGA50:50 nanoparticles exposure. Moreover, obtained data is promising to test the immunogenicity and efficacy of W-1 L19 as a nanovaccine candidate against Canine Parvovirus in mice.
